# Effectiveness of whey protein supplements on the serum levels of amino acid, creatinine kinase and myoglobin of athletes: a systematic review and meta-analysis

**DOI:** 10.1186/s13643-019-1039-z

**Published:** 2019-05-31

**Authors:** Fui-Ching Lam, Tahir Mehmood Khan, Hani Faidah, Abdul Haseeb, Amer Hayat Khan

**Affiliations:** 1 School of Pharmacy, Monash University Malaysia, Jalan Lagoon Selatan, 47500 Bandar Sunway, Selangor Darul Ehsan Malaysia; 2 The Institute of Pharmaceutical Sciences (IPS), University of Veterinary & Animal Sciences (UVAS), Outfall Road, Lahore, Pakistan; 3 College of Medicine, Umul Qura University, Makkah, Saudi Arabia; 4 College of Pharmacy, Umul Qura University, Makkah, Saudi Arabia; 5 School of Pharmaceutical Science, University Sains Malaysia, Penang, Malaysia

**Keywords:** Protein, Supplements, Evidence-based review, Sports, Performance

## Abstract

**Background:**

Consuming whey protein supplements, along with physiotherapy and psychotherapy, have been recognised in sports performance. Whey protein supplements (WPS) is one of the commonly used supplements as ergogenic aids for athletes to enhance their muscle performance and recovery during sport-related injuries. The purpose of this systematic review is to investigate the effectiveness of WPS over the blood biochemistry mainly amino acids, creatinine kinase and myoglobin which influence performance and recovery among athletes.

**Method:**

A comprehensive literature search was conducted to identify randomised control trials (RCTs) and non-RCTs that investigated the effectiveness of WPS on amino acids, creatinine kinase and myoglobin among athletes. Risk of Bias in Non-Randomised Studies of Interventions tool (ROBINS-I) and Cochrane Risk of Bias Assessment tool were used to rule out the quality of studies. Meta-analysis was performed using a random effect model with STATA version 14.2. The weighted mean difference was used to estimate the effectiveness of WPS against other supplements.

**Results:**

A total of 333,257 research articles were identified; of these, 15 records were included to proceed with the analysis. Meta-analysis has shown that WPS has significantly overall increased the level of *essential amino acids* level by 624.03 nmol/L (CI = 169.27, 1078.8; *I*^2^ = 100%; *p* = 0.00) and *branched-chain amino acids* level by 458.57 nmol/L (CI = 179.96, 737.18; *I*^2^ = 100%; *p* = 0.00) compared to the control group (without WPS). Moreover, was observed to decrease *myoglobin* level by 11.74 ng/ml (CI = − 30.24, 6.76; *I*^2^ = 79.6%; *p* = 0.007) and *creatine kinase* level by 47.05 U/L (CI = − 129.47, 35.37; *I*^2^ = 98.4%; *p* = 0.000) compared to the control group.

**Conclusion:**

The findings revealed that the clinical evidence supports the effectiveness of WPS as a positive ergogenic aid on athletes’ amino acids, creatinine kinase and myoglobin.

**Electronic supplementary material:**

The online version of this article (10.1186/s13643-019-1039-z) contains supplementary material, which is available to authorized users.

## Introduction

Athletes experience fatigue when they continuously undertake intensive physical training. Both muscular and mental fatigue assist to prevent the body from experiencing muscle damage and fracture injuries [1]. In some situations, athletes are motivated to carry on their routine exercise, regardless of fatigue [2]. This will lead them to muscle soreness which also known as delayed onset muscle soreness (DOMS) When inadequate rest and lack of care towards the DOMS, this can further lead to loss of skeletal muscle mass and induce muscle damages and fracture injuries known as sports injuries [3]. Therefore, observing creatinine kinase and myoglobin level are essential as they are biomarkers for the presence of muscle damage or inflammation after intensive exercise [4, 5].

In addition to physiotherapy sessions, athletes consume medications and supplements to boost the recovery process and performance. Often, it happened that some supplements do not disclose the presence of some illegal substances which prohibited by doping agencies—for example, anabolic androgenic steroids, diuretics and epinephrine—which can jeopardise athletes’ careers as they may face penalties or be removed from competitions [6]. Moreover, due to the lack of quality control, some supplements might contain some substance that is prohibited, or in some case, the concentration of that specific substance may be higher than the allowed dose or limits. In some cases, these substances lead to additional complications that prolong the recovery process and mean opportunities to participate in competitions are lost [7].

The World Anti-Doping Agency (WADA) is cautious in supplementation consumption among athletes. A WADA-accredited laboratory examined 600 nutritional supplements and found that approximately 15% (%) contained anabolic steroids, which was not disclosed on the bottle label, packaging or leaflets [8]. One of the most widely used supplements adopted to the WADA recommendations is WPS [9]. Whey protein has had a large impact on nutritional supplements for the community especially athletes as it contains nearly 50% of essential amino acids (EAA) and about 26% of branched-chain amino acids (BCAA). Moreover, the amino acid composition provided by whey protein has a similar pattern to human skeletal muscle amino acid composition, so it is absorbed more rapidly than other protein sources [1]. About 60% of the protein can stimulate skeletal muscles in the human body [10]. Moreover, whey protein can reduce fatigue augmenting muscle protein synthesis and slightly suppresses muscle protein breakdown [11]. To date, there are few systematic reviews that have explored the impact of whey protein on the body composition and resistance workout-induced improvements in muscle mass and strength [12, 13]. However, there is hardly any systematic evidence that investigate the effectiveness of WPS over the blood biochemistry mainly amino acids, creatinine kinase, and myoglobin which influence performance and recovery among athletes. The current systematic review will specifically look into this aspect of WPS and aim to statistically rule out the effect of WPS on the blood biochemistry; amino acids, creatinine kinase and myoglobin of athletes.

## Methods

A systematic review was conducted to investigate the effectiveness of WPS over the blood biochemistry mainly amino acids, creatinine kinase and myoglobin which influence performance and recovery among athletes. Preferred Reporting Items for Systematic Reviews and Meta-Analyses (PRISMA) were used to perform the systematic search [14] A protocol of this systematic review is registered in PROSPERO 2016 [CRD42016041842] [15].

### Search terms and search strings

The search strategy used the keyword of “whey*” combined individually with “athlete*”, “injury*”, “muscle*”, “perform*” and “recover*” to find relevant articles from the databases [16]. Thesaurus terms were applied to medical databases such as PubMed and EMBASE, which were Medical Subject Headings (MeSH) and Embase Subject Headings (EMTREE) [17].

Proper care was taken to remove the error by resetting filters. For instance, the PubMed database has a filtering function for selected species of human or animal. When filtered on animal species’ studies, studies examined on humans were found, as the WP could originate from cow’s milk. Therefore, when filtered on human species only, studies categorised under the animal species that examined humans may have been omitted. Hence, the databases’ filtering or customising functions were not used as the function would eliminate relevant articles.

### Databases selected

Comprehensive literature search was done across medical and health science database such as PubMed, EMBASE via Ovid, Scopus, Cochrane, Cumulative Index to Nursing and Allied Health Literature (CINAHL) via EBSCOhost, SPORTDiscus, Health & Medicine Database via ProQuest, Wiley Online Library, Web of Science, ScienceDirect, Taylor & Francis and SAGE. Manual searches in bibliographies of relevant review articles were also performed to identify any other paper that was not indexed in the selected databases. In addition, all the sport-related journals were individually searched for any potential paper that might meet the inclusion criteria.

### Inclusive studies design

Inclusive studies design for the systematic review was randomised controlled trials (RCTs) and non-RCTs designs. No restriction was placed on language. The searched timeframe was from the inception of the databases until 31 January 2017. However, study designs on expert opinions, case reports/series, surveys, review articles, editorials, commercial advertisements, magazine articles, unpublished articles and theses were excluded.

### Population intervention comparator and outcomes (PICO)

#### Population

The population includes active athletes who experienced fatigue and had recovered and/or been hindered in their performance. Studies observed on retired athletes, mixed athletes with non-athletes, animals, cells and gels were excluded.

#### Intervention

The interventions include whey protein or supplements containing whey protein. The intervention can be found in the form of isolate, concentrate, hydrolysate, denature and protein bars.

#### Comparator

The comparators were carbohydrate supplements, protein-containing foods from animal sources (e.g., meat, fish, dairy products, and eggs), protein-containing vegetarian sources (e.g., tofu, legumes, and soy protein), vitamins (e.g., multivitamin, vitamin B, beta-carotene, and folic acid), minerals (e.g., calcium, iron and zinc) and placebos (include no treatment and treatment as usual).

#### Outcomes

The outcome of interest observed is the effect of WPS amino acids, creatinine kinase and myoglobin.

### Conducting the search and selection process

The relevant articles were compiled, and duplicate articles were removed by using EndNote X7. Then, a screening was done on titles and abstracts of the relevant articles based on the inclusion and the exclusion criteria. After that, full-text articles of the screened articles were retrieved. However, in some cases where data was presented as conference abstracts or some additional clarification regarding the data was required, corresponding authors of the specific paper were contacted for further assistance. All the data extraction sheets were piloted, and extraction of all papers was performed by TMK and FCL individually. If there were any variations in the extractions, were resolved by the mutual consensus.

### Data extraction

The extracted data was entered into Microsoft Excel 2016, namely [18]

1. General information (first author surname, title, and year of publication, journal name)
2. The article study methods and characteristic (study design)
3. Participants (age, gender, weight, heights and sporting activity)
4. Intervention (dose and number times consumed)
5. Comparators (dose and number times consumed)

The outcome is the data obtained after the participants consumed the intervention or control on amino acids, creatinine kinase and myoglobin. Most of the data are located within the text of the articles and presented in tabular form or graphs. When data was in standard error or standard error mean, it was transformed into a standard deviation [19].

### Assessment of risk of bias for included studies

The inclusive studies were assessed for risk of bias (RoB) by two reviewers independently. Both assessment results were compared and verified for accuracy. A Cochrane Risk of Bias tool criteria was used to assess the RCT studies [20]. For non-RCT studies, the RoB was assessed using Cochrane Risk of Bias Table and Risk of Bias in Non-Randomised Studies of Interventions tool (ROBINS-I), comparing two or more interventions and presenting a judgement. The ROBINS-I was an upgraded version of the Cochrane Risk of Bias Assessment Tool: for Non-Randomised Studies of Interventions (ACROBAT-NRSI).

### Data synthesis

Meta-analysis was performed using a random effect model with STATA version 14.2. The type of data for this analysis was continuous data, which contained mean, standard deviation and sample size [21]. A random effect model was selected since there were no identical studies throughout all the included studies and the participants were various categories of athletes, which could have had an impact on the intervention effect [22]. For the meta-analysis arm, the intervention was considered the experimental arm while control arms were alternative supplements or proteins with equivalent quantity and similar visuals such as carbohydrate, placebo, maltodextrin and bovine colostrum. The outcomes were on EAA, BCAA, creatinine kinase and myoglobin.

Two or more eligible studies for an outcome were required to generate weighted mean differences (WMD), 95%confidence intervals (CI), weight percentage, heterogeneity chi-squared, *I*-squared (*I*^2^) for variation in WMD attributable to heterogeneity, Tau-squared to estimate between-study variance, and forest plot. WMD was preferred as outcome measurements in all studies were made on the same scale [23]. When the *I*-square appeared to have more than 50% of heterogeneity, subgroup meta-analyses were conducted by activities or exercises instructed during the study and intervention duration (days). Funnel plots and Egger tests were also computed to examine publication bias.

## Results

### Inclusive articles selection

**Fig. 1:**
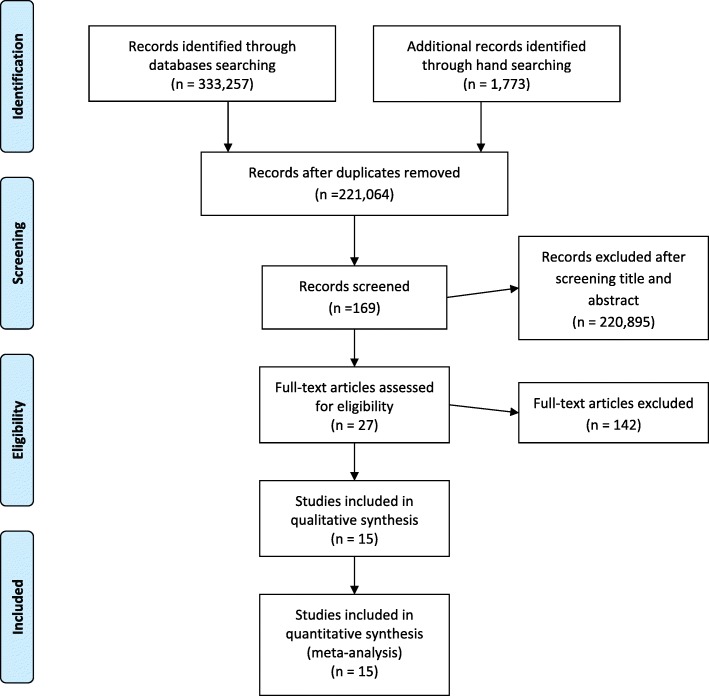
PRISMA flow chart

For the identified articles, there were 333,257 records from the databases and 1773 records through a manual search. At the screening stage, there were 221,064 records after removing duplicates from the identified stage. After screening the titles and abstract, 169 records were brought to the next stage. Subsequently, 27 records were eligible, as 147 records were excluded given the reading availability of the full text of the articles. Of these 27 papers, 15 papers were found addressing the clinical parameters described in the objectives of this systematic review. The PRISMA flow of these stages is shown in Fig. 1.

### Study characteristics

The descriptive study characteristics are presented in Table 1. Of these studies, 13 studies were RCTs [26, 31–33, 38] with crossover [35–37], blocking [27, 28], placebo control, counterbalanced [24, 25, 29] study designs. On the other hand, two studies were non-RCTs with crossover double blinding [34] and counterbalanced within-group double blinding [30] study design. The total number of participants was 230, with 207 males and 23 females. Only four studies included both genders. The number of participants ranged from 8 to 24. The participants were from different sports: soccer, badminton, cycling, elite orienting and people from track and field. The intervention duration was from 1 day to 60 days.

**Table 1:** Summary of the characteristic of the all-inclusive studies

Author name and year	Country	Study design	Participants				Protocol				Outcomes assessed
			Categories of athletes	Male	Female	Total	Number of times to obtain supplement in a day	Intervention duration (day)	Exercise activity during intervention duration	Consume the supplementation during intervention duration	
[24]	Australia	Random within-subject, counterbalanced	Young, healthy, resistance-trained	8	7	15	1 time(s)	60 days, include wash out period	Leg press	Consume the supplementation during exercise	Essential amino acid, branched-chain amino acids
[25]	Spain	Random counterbalanced double blinding	Cyclists	15	0	15	1 time(s)	16 days	Cycling	Consume the supplementation during exercise	Creatine kinase
[26]	Denmark	Random	Soccer players	16	0	16	1 time(s)	2 days	Soccer game	Consume the supplementation after exercise	Myoglobin, creatine kinase
[27]	Denmark	Random block single blinding	Elite orienteers	8	10	18	1 time(s)	7 days	Running	Consume the supplementation before and after exercise	Creatine kinase
[28]	Denmark	Random block single blinding	Elite orienteers	18	0	18	4 time(s)	7 days	Cycling, mix of distance training, interval training, mountain climbing	Consume the supplementation after exercise	Creatine kinase
[29]	The UK	Random counterbalanced	Cyclists and tri-athletes	9	0	9	1 time(s)	7 days	Cycling	Consume the supplementation before exercise	Essential amino acid, branched-chain amino acids
[30]	The USA	Non-random counterbalanced within-group double blinding	Resistance training experience	13	0	13	2 time(s)	56 days, include wash out period	Cycle, dynamic stretches, jump test	Consume the supplementation before and during exercise	Creatine kinase
[31]	Indonesia	Random double blinding	Badminton player	18	0	18	1 time(s)	4 days	Resistance exercise was conducted using squat	Consume the supplementation after exercise	Creatine kinase
[32]	Brazil	Random double blinding	Soccer players	24	0	24	1 time(s)	56 days	Cycle, soccer	Consume the supplementation after exercise	Creatine kinase
[33]	Brazil	Random	Soccer players	24	0	24	2 time(s)	180 days	Soccer training	Consume the supplementation before exercise and after exercise	Creatine kinase
[34]	Japan	Non-random crossover double blinding	Trained men	8	0	8	4 time(s)	9 days, include wash out period	Cycle	Consume the supplementation after exercise	Branched-chain amino acids
[35]	The UK	Random counterbalanced, crossover double blinding	Amateur soccer players	16	0	16	4 time(s)	13 days, include wash out period	Run, jogging, running	Consume the supplementation before, during and after exercise	Myoglobin, creatine kinase
[36]	Australia	Random counterbalanced, crossover	Physically active	8	0	8	2 time(s)	16 days, include wash out period	Plate-loaded leg extension	Consume the supplementation after exercise	Essential amino acid, branched-chain amino acids
[37]	Canada	Random crossover, counterbalanced double blinding	Resistance-trained	8	0	8	1 time(s)	16 days	Resistance exercise, weight lifted	Consume the supplementation before exercise	Essential amino acid, branched-chain amino acids
[38]	China	Randomised control trial	Track and field athletes	14	6	20	3 time(s)	1 days	Track and field	Consume the supplementation before and after exercise	Creatine kinase

### Risk of bias

**Fig. 2:**
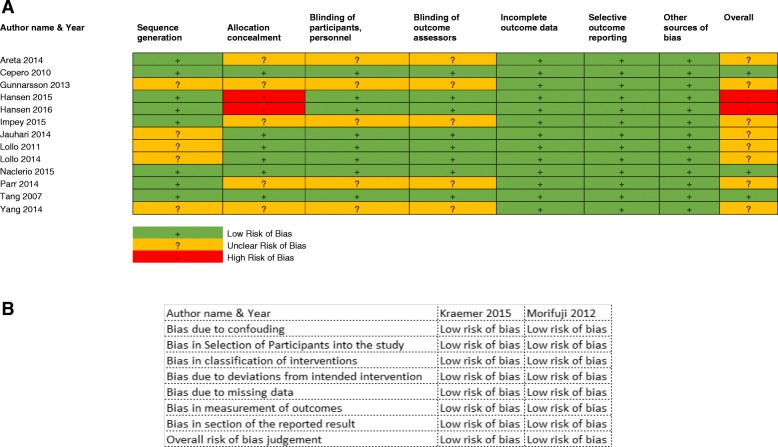
Summary Cochrane ROB assessment for individual RCTs studies (**a**) and summary of ROBINS-I assessment for individual non-RCTs studies (**b**)

A total of 13 RCT studies were assessed using the Cochrane RoB assessment (see online Additional file 1: Table S1). The domains and overall assessment are shown in Fig. 2a. This illustrates that all the studies had a low RoB in “incomplete outcome data” and “other sources of bias”. Eight studies had at least one domain of unclear RoB in “sequence generation”, “allocation concealment”, “blinding of participants and personnel” and “blinding of outcome assessors”. Two studies had high RoB for either “allocation concealment” or “selective outcome reporting” [27, 28]. On the other hand, two non-RCTs studies were assessed (see online Additional file 2: Table S2) based on ROBINS-I, as shown in Fig. 2b, which had a low RoB in all domains.

### Meta-analysis

A random effect model of a meta-analysis of 15 studies was conducted to investigate the effectiveness of WPS as compared to other supplements for amino acids, creatinine kinase and myoglobin. Although the studies found at overall of high RoB, they were not removed from the meta-analysis.

### Amino acids

**Fig. 3:**
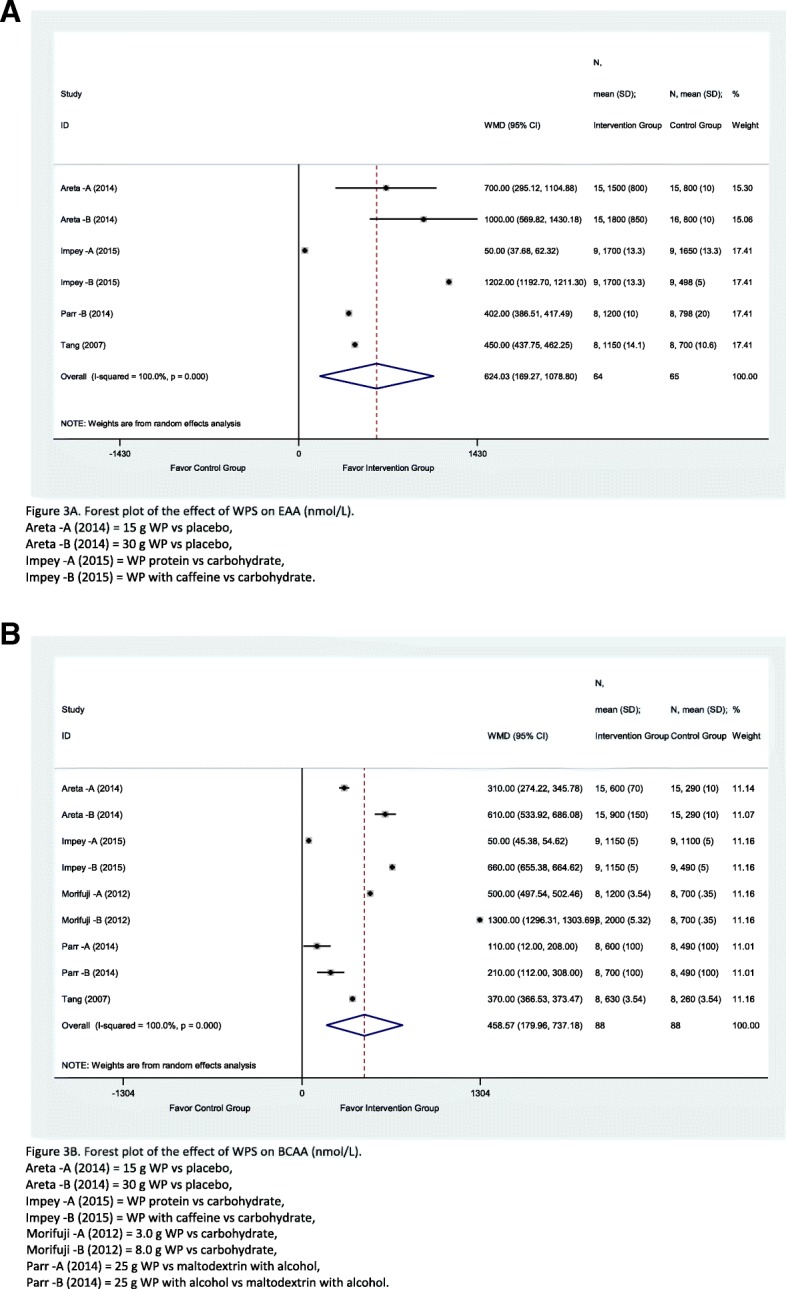
A forest plot of the effect of WPS on EAA (nmol/L). Forest plot of meta-analysis on EAA (**a**) and BCAA (**b**)

A total of six studies have investigated the outcome of WPS over the EAA, and nine studies reported the outcome relevant to BCAA. Overall, it is seen that WPS manage to induce EAA levels among the groups of athletes consuming WPS during the intervention of study period 624.03 nmol/L (CI = 169.27, 1078.8; *I*^2^ = 100%; *p* = 0.00) compared to the control groups, although high heterogeneity was detected (Fig. 3a). The individual studies were all favourable to the intervention and their weighted influence of the individual studies was similarly distributed. Similarly, the effect of WPS on BCAA level was significantly better in the intervention group than the control group by 458.57 nmol/L (CI = 179.96, 737.18; *I*^2^ = 100%; *p* = 0.00) and all studies were favourable to the intervention group (Fig. 3a). The weighted influence of all the individual studies was equally distributed by 11%. However, both of the outcomes had high heterogeneity between studies, with an *I*^2^ of 100%, which can be mainly due to the diversity in the number of respondents and level of effect which was varying from one study to another. Furthermore, the overall subgroup analyses of EAA and BCAA are merely explained about the heterogeneity as the *I*^2^ value remained high and a standalone study (see online Additional file 3: Table S3).

### Myoglobin

**Fig. 4:**
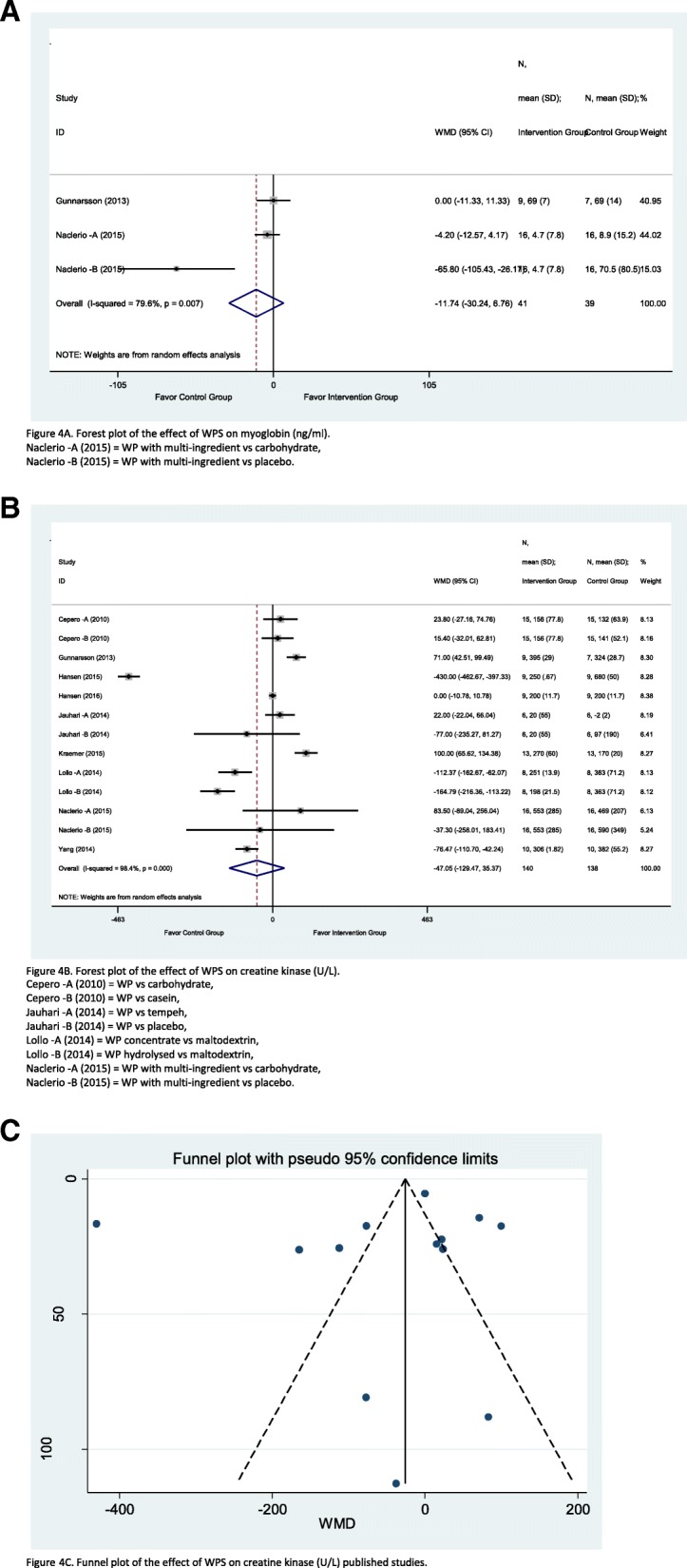
Forest plot of meta-analysis on myoglobin (**a**), creatine kinase (**b**), funnel plot for the studies estimating the effect of whey protein over creatine kinase (**c**)

Three studies were found involved and exploring the effect WPS with myoglobin. Figure 4a illustrates that the overall WMD of myoglobin level reduces in the intervention group by 11.74 ng/ml (CI = − 30.24, 6.76; *I*^2^ = 79.6%; *p* = 0.007) compared to the control group, yet it has moderate–high heterogeneity. Two studies were favourable to the control group: Naclerio et al.—A [35] (weighted = 44.02%) and Naclerio et al.—B [35] (weighted = 15.03%), while the Gunnarsson et al. [26] study lie on the no effect line and had the highest weighted influence amount of 40.95%. However, the subgroup analyses did not explain the heterogeneity as the *I*^2^ value remained high and a standalone study (see online Additional file 3: Table S3).

### Creatinine kinase

A total of thirteen studies involved WPS with creatinine kinase. Figure 4b illustrates that the overall creatinine kinase levels were 47.05 U/L (CI = − 129.47, 35.37; *I*^2^ = 98.4%; *p* = 0.000) significantly lower in the intervention group than in the control group, although with high heterogeneity. Six studies were also favourable to the intervention group: the Gunnarsson et al. [26] study carried the highest (8.30%) weighted influences and Naclerio et al.—A [35] study carried the lowest (6.13%) weighted influences. Also, six studies were favourable to the control group: Hansen et al. [27] study carried the highest (8.28%) weighted influences and Naclerio et al.—B [35] study carried the lowest (5.24%) weighted influences. The Hansen et al. [28] study is the only study that lies on the no effect line with a weighed influence of 8.38%. For the publication bias, the funnel plot depicts that there was publication bias as the majority of studies were away from average and outside of the 95% confidence limits (Fig. 4c), along with the Egger test (see online Additional file 3: Table S3), where the bias was − 2.1 (CI = − 9.96, 5.75; *p* = 0.567).

For the subgroup analyses, the physical activities analysis (see online Additional file 3: Table S3) shows that the cycle group had no heterogeneity (*I*^2^ = 0%; CI = − 15.42, 54.01) and the resistance exercise subgroup had low evidence and heterogeneity (*I*^2^ = 28.3%; CI = − 73.71, 79.47). However, the soccer, run, cycle and resistance subgroups have high heterogeneity of 95% and above in *I*^2^. On the other hand, the heterogeneity for the exercise resistance group was found to be 28.3%. In the intervention duration range (see online Additional file 3: Table S3), the differences between all subgroups are statistically insignificant. The range period of 1–20 days has high heterogeneity of 98.7% in *I*^2^, whereas the range of 161–180 days has moderate–low heterogeneity (*I*^2^ = 50%), and the range of 41–60 days was a stand-alone study (see online Additional file 3: Table S3).

## Discussion

This is perhaps the first systematic review and meta-analysis to investigate the effectiveness of WPS over the blood biochemistry mainly amino acids, creatinine kinase and myoglobin which influence performance and recovery among athletes. Then again, the intervention was described as WPS, while others as comparators. The search strategy was robust and unlikely to have missed eligible studies. Of the collected studies, 13 (96%) of the included studies were RCTs which many sources of bias had removed from the process [23]. Two non-RCTs are high quality and the overall assessments had low RoB; this indicated that the two non-RCTs are comparable to RCTs. Meta-analysis is a statistical measurement and procedure for combining data from the multiple studies and developed a statistically single conclusion. The purposes of the meta-analysis are precisely estimate the effect magnitude and identify the reason for the variation and common effect and outcome of data [39].

Whey protein supplements having high levels of serum amino acids of both EAA and BCAA are well known. Furthermore, the results of the meta-analyses illustrated robust evidence that athletes who consumed WPS had higher levels of serum amino acids than comparators. Essential amino acids of WPS were believed to retain and growth of muscle, while BCAA of WPS was believed to delay the onset of fatigue during prolonged endurance exercise [40–42]. Moreover, Areta et al. [24] investigated that amino acids of WPS support muscle protein while Impey et al. [29] examined WPS enhanced post-exercise muscle protein synthesis rates. Tang et al. [37] also investigated that a small dose of WP (10 g) was able to stimulate muscle protein synthesis athletes after exercise. Therefore, serum amino acid from WPS absolute ergogenic benefits athletes on delay and recovery from the sports injuries and fatigue [40, 43].

In addition, the myoglobin and creatinine kinase levels were lower in the intervention group which indicates that the consumption of WPS can reduce the muscle fatigue or muscle damage than the comparator groups. The release and elevation in myoglobin indicates the presence of muscle damage or inflammation after exercise [4]. Thus, myoglobin acts as a blood marker for muscle damage [44]. Moreover, kidneys can be impaired when extreme levels of myoglobin are released, known as rhabdomyolysis [45]. Subsequently, a lower level of myoglobin would diminish muscle fatigue to prevent muscle damage while athletes drive their strength [4]. According to the meta-analysis, the overall myoglobin level in the intervention group was lower than that in the control group. Surprisingly, studies have shown that consuming WPS seems to have ergogenic aids as it does lower the myoglobin level [26, 35]. Subsequently, a lower level of myoglobin was reflected in athletes’ physical effort: they could go beyond their maximum physical strength while preventing any severe muscle damage [44].

Creatinine kinase appearing in the blood is considered as a marker of indirect muscle damage [5]. The level is used to assist in detecting athletes’ body condition of tissue damage. It is reasonable for the creatinine kinase level to elevate temporarily due to strenuous exercise [25, 28], but the level should not rise to an extent that could damage skeletal muscles, heart or brain [46]. Therefore, it is essential for athletes to have a lower creatinine kinase level while driving their physical strength. Based on studies, consuming of WPS does lower creatinine kinase level for active athletes [31, 33]. Moreover, Kraemer et al. [30] observed that the WPS delay muscle soreness and improve the intensity of the physical performance. Lollo et al. [33] also studied that the positive effect of WPS on attenuated creatine kinase level could be because the properties of WPS have antioxidant capacity. Hence, lower creatine kinase when consuming WPS will aid athletes to prolong time to fatigue and better maintain or improve exercise performance.

Based on evidence and analyses, WPS is found to be effective in improving the serum levels of BCAA and EAA, and on other hand, WPS has shown a substantial effect on reducing myoglobin and creatinine kinase levels that are markers of preventing sports injuries, These result support the consumption of WPS for the athletes during the routine training and muscle injuries to augment the muscle performance and recovery process.

### Limitation

However, there are two main concerns that researchers would like to highlight before any athlete and multidisciplinary team who manages athletes’ health and performance should opt to use WPS; the first one is the higher level of heterogeneity across the compared studies. The subgroup analysis was performed which has shown some declined in heterogeneity for some specific groups. However, for some groups, higher heterogeneity was still there, which is one of the genuine concerns for the researchers while interpreting the results of this meta-analysis. Moreover, the difference in WPS formulation also might have affected the bioavailability and outcome among the studies, and this clinical aspect might have contributed to the heterogeneity in the current meta-analysis.

### Recommendation

Future directions for research and conducting research include larger sample sizes, the inclusion of both genders (especially on female athletes), ages, geographical, type of sport and categories of athletes. Interventions that are consumed before, during and/or after sports performances and recovery process also deserve further study, considering the effectiveness of improving athletes’ sports performances and recovery. Additionally, follow-up studies could establish effectiveness for the relation between interventions and long-term performance recovery progress for athletes.

Nonetheless, although WP is recognised as safe supplements for athletes [47, 48], concern arises from WADA insight whereby illegal substances can be found in the interventions from the included studies. Two studies reported an intervention containing caffeine [26, 29] and a study had an intervention containing alcohol [36].

The WADA guidelines and recommendations are updated annually and serve as a guide for consuming supplements during the supports and recovery process for athletes. Therefore, it is highly recommended for athletes, and the multidisciplinary team are well-informed and updated themselves on the guidelines and recommendations before using WPS or any supplements.

## Conclusion

In conclusion, the current meta-analysis shows the effectiveness of WPS over the blood biochemistry mainly amino acids, creatinine kinase and myoglobin which influence the performance and recovery among athletes and are promising. First of all, the quality of studies has delivered assurance in the validity and reliability of the clinical evidence, whereby most of all the studies were RCTs and, thus, many sources of biases have been omitted. Included studies examined the conditions as close to real life training and competition conditions as possible for athletes. Importantly, athletes need to check, maintain and control the dose as set out by WADA. Moreover, the positive impact of WPS on the essential biomarkers (myoglobin and creatine kinase) aids athletes by delaying or attenuating fatigue and reducing the risk of sports injuries while athletes are reaching beyond their potential aerobic threshold.

## Additional files

Additional file 1: Risk of Bias. (XLSX 21 kb)

Additional file 2: The Risk Of Bias In Non-randomized Studies – of Interventions (ROBINS-I) assessment tool. (DOCX 107 kb)

Additional file 3: Meta-analysis output. (ZIP 162 kb)
