# Supplementary material for: Effectiveness of whey protein supplements on the serum levels of amino acid, creatinine kinase and myoglobin of athletes: a systematic review and meta-analysis
Source: Syst Rev. 2019 May 31;8:130. doi: 10.1186/s13643-019-1039-z (PMC6544960; doi:10.1186/s13643-019-1039-z)
Supplement: Supplementary file 2 — The Risk Of Bias In Non-randomized Studies – of Interventions (ROBINS-I) assessment tool. (DOCX 107 kb) [file 13643_2019_1039_MOESM2_ESM.docx]

The Risk Of Bias In Non-randomized Studies – of Interventions (ROBINS-I) assessment tool

**Version 19 September 2016**

Article title: The addition of beta-hydroxy-beta-methylbutyrate and isomaltulose to whey protein improves recovery from highly demanding resistance exercise

Authors: Kraemer, William J.; Hooper, David R.; Szivak, Tunde K.; Kupchak, Brian R.; Dunn-Lewis, Courtenay; Comstock, Brett A.; Flanagan, Shawn D.; Looney, David P.; Sterczala, Adam J.; DuPont, William H.; Pryor, J. Luke; Luk, Hiu-Ying; Maladoungdock, Jesse; McDermott, Danielle; Volek, Jeff S.; Maresh, Carl M.


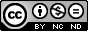


This work is licensed under a [Creative Commons Attribution-NonCommercial-NoDerivatives 4.0 International License](http://creativecommons.org/licenses/by-nc-nd/4.0/).

# ROBINS-I tool (Stage I): At protocol stage

## Specify the review question

| Participants | Athletes experience recovering from injuries and/or hinder in performance. |
| --- | --- |
| Experimental intervention | Whey protein or the whey supplements. It can be in form of isolate, concentrate, or hydrolysate. |
| Comparator | Carbohydrate supplement; protein-containing foods include animal sources, and vegetarian sources; vitamins; minerals; placebo |
| Outcomes | level of the protein in blood and creatine kinase activity; development of muscle and bone tissue; the muscle and bone fracture injuries recovery period; pain level; and mobility, strength and performance level |

## List the confounding domains relevant to all or most studies

| Consume whey protein |
| --- |

## List co-interventions that could be different between intervention groups and that could impact on outcomes

| None |
| --- |

# ROBINS-I tool (Stage II): For each study

## Specify a target randomized trial specific to the study

| Design | Matched (Counterbalanced within-group) |
| --- | --- |
| Participants | Participants who have resistance training experience |
| Experimental intervention | whey protein (100 kcal, 20 g protein, 2.5 g carbohydrate, 1 g fat) |
| Comparator | RP supplement (260 kcal, 20 g protein, 1.5 g HMB, 41 g carbohydrate, 2 g fat) |

## Is your aim for this study…?

| ☑ | to assess the effect of *assignment to* intervention |
| --- | --- |
| □ | to assess the effect of *starting and adhering to* intervention |

## Specify the outcome

Specify which outcome is being assessed for risk of bias (typically from among those earmarked for the Summary of Findings table). Specify whether this is a proposed benefit or harm of intervention.

| Plasma insulin-like growth factor I, Creatine Kinase, Cortisol and Testosterone |
| --- |

## Specify the numerical result being assessed

In case of multiple alternative analyses being presented, specify the numeric result (e.g. RR = 1.52 (95% CI 0.83 to 2.77) and/or a reference (e.g. to a table, figure or paragraph) that uniquely defines the result being assessed.

| Table 2 Hormonal Response Data and Subject Characteristics |
| --- |

## Preliminary consideration of confounders

Complete a row for each important confounding domain (i) listed in the review protocol; and (ii) relevant to the setting of this particular study, or which the study authors identified as potentially important.

#### “Important” confounding domains are those for which, in the context of this study, adjustment is expected to lead to a clinically important change in the estimated effect of the intervention. “Validity” refers to whether the confounding variable or variables fully measure the domain, while “reliability” refers to the precision of the measurement (more measurement error means less reliability).

| **(i) Confounding domains listed in the review protocol** | | | | |
| --- | --- | --- | --- | --- |
| Confounding domain | Measured variable(s) | Is there evidence that controlling for this variable was unnecessary?* | Is the confounding domain measured validly and reliably by this variable (or these variables)? | OPTIONAL: Is failure to adjust for this variable (alone) expected to favour the experimental intervention or the comparator? |
| Not Applicable (NA) |  |  | Yes / No / No information | Favour experimental / Favour comparator / No information |
|  |  |  |  |  |
|  |  |  |  |  |
|  |  |  |  |  |
|  |  |  |  |  |
|  |  |  |  |  |
|  |  |  |  |  |
|  |  |  |  |  |
|  |  |  |  |  |

| **(ii) Additional confounding domains relevant to the setting of this particular study, or which the study authors identified as important** | | | | |
| --- | --- | --- | --- | --- |
| Confounding domain | Measured variable(s) | Is there evidence that controlling for this variable was unnecessary?* | Is the confounding domain measured validly and reliably by this variable (or these variables)? | OPTIONAL: Is failure to adjust for this variable (alone) expected to favour the experimental intervention or the comparator? |
| NA |  |  | Yes / No / No information | Favour experimental / Favour comparator / No information |
|  |  |  |  |  |
|  |  |  |  |  |
|  |  |  |  |  |
|  |  |  |  |  |
|  |  |  |  |  |
|  |  |  |  |  |
|  |  |  |  |  |
|  |  |  |  |  |
|  |  |  |  |  |

* In the context of a particular study, variables can be demonstrated not to be confounders and so not included in the analysis: (a) if they are not predictive of the outcome; (b) if they are not predictive of intervention; or (c) because adjustment makes no or minimal difference to the estimated effect of the primary parameter. Note that “no statistically significant association” is not the same as “not predictive”.

## Preliminary consideration of co-interventions

Complete a row for each important co-intervention (i) listed in the review protocol; and (ii) relevant to the setting of this particular study, or which the study authors identified as important.

#### “Important” co-interventions are those for which, in the context of this study, adjustment is expected to lead to a clinically important change in the estimated effect of the intervention.

| **(i) Co-interventions listed in the review protocol** | | |
| --- | --- | --- |
| Co-intervention | Is there evidence that controlling for this co-intervention was unnecessary (e.g. because it was not administered)? | Is presence of this co-intervention likely to favour outcomes in the experimental intervention or the comparator |
| NA |  | Favour experimental / Favour comparator / No information |
|  |  | Favour experimental / Favour comparator / No information |
|  |  | Favour experimental / Favour comparator / No information |
|  |  | Favour experimental / Favour comparator / No information |

| **(ii) Additional co-interventions relevant to the setting of this particular study, or which the study authors identified as important** | | |
| --- | --- | --- |
| Co-intervention | Is there evidence that controlling for this co-intervention was unnecessary (e.g. because it was not administered)? | Is presence of this co-intervention likely to favour outcomes in the experimental intervention or the comparator |
| NA |  | Favour experimental / Favour comparator / No information |
|  |  | Favour experimental / Favour comparator / No information |
|  |  | Favour experimental / Favour comparator / No information |
|  |  | Favour experimental / Favour comparator / No information |

## Risk of bias assessment

Responses underlined in green are potential markers for low risk of bias, and responses in red are potential markers for a risk of bias. Where questions relate only to sign posts to other questions, no formatting is used.

|  | **Signalling questions** | **Description** | **Response options** |
| --- | --- | --- | --- |
| **Bias due to confounding** | | | |
|  | 1.1 Is there potential for confounding of the effect of intervention in this study?  **If N/PN to 1.1:** the study can be considered to be at low risk of bias due to confounding and no further signalling questions need be considered | - Before supplement loading, subjects were asked to complete a trial 3-day diet record, which served as a familiarization. - Subjects were instructed to follow the prescription during the subsequent 2-week supplement loading phase. | N |
|  | **If Y/PY to 1.1**: determine whether there is a need to assess time-varying confounding: |  |  |
|  | 1.2. Was the analysis based on splitting participants’ follow up time according to intervention received?  **If N/PN**, answer questions relating to baseline confounding (1.4 to 1.6)  **If Y/PY**, go to question 1.3. |  |  |
|  | 1.3. Were intervention discontinuations or switches likely to be related to factors that are prognostic for the outcome?  **If N/PN**, answer questions relating to baseline confounding (1.4 to 1.6)  **If Y/PY**, answer questions relating to both baseline and time-varying confounding (1.7 and 1.8) |  |  |

|  | **Questions relating to baseline confounding only** | | |
| --- | --- | --- | --- |
|  | 1.4. Did the authors use an appropriate analysis method that controlled for all the important confounding domains? |  |  |
|  | 1.5. **If Y/PY to 1.4**: Were confounding domains that were controlled for measured validly and reliably by the variables available in this study? |  |  |
|  | 1.6. Did the authors control for any post-intervention variables that could have been affected by the intervention? |  |  |
|  | **Questions relating to baseline and time-varying confounding** | |  |
|  | 1.7. Did the authors use an appropriate analysis method that controlled for all the important confounding domains and for time-varying confounding? |  |  |
|  | 1.8. **If Y/PY to 1.7**: Were confounding domains that were controlled for measured validly and reliably by the variables available in this study? |  |  |
|  | **Risk of bias judgement** |  | Low |
|  | Optional: What is the predicted direction of bias due to confounding? |  |  |

| **Bias in selection of participants into the study** | | | |
| --- | --- | --- | --- |
|  | 2.1. Was selection of participants into the study (or into the analysis) based on participant characteristics observed after the start of intervention?  **If N/PN to 2.1:** go to 2.4 | All subjects were fully informed of the protocol design and associated risks of this investigation before signing an informed consent approved by the University of Connecticut Institutional Review Board for use of human subjects. | N |
|  | 2.2. **If Y/PY to 2.1**: Were the post-intervention variables that influenced selection likely to be associated with intervention?  2.3 **If Y/PY to 2.2**: Were the post-intervention variables that influenced selection likely to be influenced by the outcome or a cause of the outcome? |  |  |
|  | 2.4. Do start of follow-up and start of intervention coincide for most participants? | No dropout or withdraw. 100% full participants | Y |
|  | 2.5. **If Y/PY to 2.2 and 2.3, or N/PN to 2.4**: Were adjustment techniques used that are likely to correct for the presence of selection biases? |  |  |
|  | **Risk of bias judgement** |  | Low |
|  | Optional: What is the predicted direction of bias due to selection of participants into the study? |  |  |

| **Bias in classification of interventions** | | | |
| --- | --- | --- | --- |
|  | 3.1 Were intervention groups clearly defined? | Thirteen men (age: 22.6 ± 3.9 years; height: 175.3 ± 12.2 cm; weight: 86.2 ± 9.8 kg) with at least one year of resistance training experience volunteered to participate in the study. Height was measured using a stadiometer (Seca, Hamburg, Germany). Weight was measured using a calibrated scale (OHAUS Corp., Florham Park, NJ). | Y |
|  | 3.2 Was the information used to define intervention groups recorded at the start of the intervention? | Same as above | Y |
|  | 3.3 Could classification of intervention status have been affected by knowledge of the outcome or risk of the outcome? | Collection of the information at the time is sufficient | N |
|  | **Risk of bias judgement** |  | Low |
|  | Optional: What is the predicted direction of bias due to classification of interventions? |  |  |

| **Bias due to deviations from intended interventions** | | | |
| --- | --- | --- | --- |
|  | **If your aim for this study is to assess the effect of assignment to intervention, answer questions 4.1 and 4.2** | |  |
|  | 4.1. Were there deviations from the intended intervention beyond what would be expected in usual practice? | Crossover study. Each participate receive both supplements. Fig. 1 A | N |
|  | 4.2. **If Y/PY to 4.1**: Were these deviations from intended intervention unbalanced between groups *and* likely to have affected the outcome? |  |  |
|  | **If your aim for this study is to assess the effect of starting and adhering to intervention, answer questions 4.3 to 4.6** | |  |
|  | 4.3. Were important co-interventions balanced across intervention groups? |  |  |
|  | 4.4. Was the intervention implemented successfully for most participants? |  |  |
|  | 4.5. Did study participants adhere to the assigned intervention regimen? |  |  |
|  | 4.6. **If N/PN to 4.3, 4.4 or 4.5**: Was an appropriate analysis used to estimate the effect of starting and adhering to the intervention? |  |  |
|  | **Risk of bias judgement** |  | Low |
|  | Optional: What is the predicted direction of bias due to deviations from the intended interventions? |  |  |

| **Bias due to missing data** | | | |
| --- | --- | --- | --- |
|  | 5.1 Were outcome data available for all, or nearly all, participants? | Outcomes based on all the participants | Y |
|  | 5.2 Were participants excluded due to missing data on intervention status? | No participants were excluded | N |
|  | 5.3 Were participants excluded due to missing data on other variables needed for the analysis? | No participants were excluded | N |
|  | 5.4 **If PN/N to 5.1, or Y/PY to 5.2 or 5.3**: Are the proportion of participants and reasons for missing data similar across interventions? |  |  |
|  | 5.5 **If PN/N to 5.1, or Y/PY to 5.2 or 5.3**: Is there evidence that results were robust to the presence of missing data? |  |  |
|  | **Risk of bias judgement** |  | Low |
|  | Optional: What is the predicted direction of bias due to missing data? |  |  |

| **Bias in measurement of outcomes** | | | |
| --- | --- | --- | --- |
|  | 6.1 Could the outcome measure have been influenced by knowledge of the intervention received? | informed consent approved by the University of Connecticut Institutional Review Board for use of human subjects and do not involve negligible assessor judgment | N |
|  | 6.2 Were outcome assessors aware of the intervention received by study participants? | It is double blinded study design | N |
|  | 6.3 Were the methods of outcome assessment comparable across intervention groups? | Both group outcomes are using the same assessment | Y |
|  | 6.4 Were any systematic errors in measurement of the outcome related to intervention received? | The method impose are well explain for conducting the study and blinded | N |
|  | **Risk of bias judgement** |  | Low |
|  | Optional: What is the predicted direction of bias due to measurement of outcomes? |  |  |

| **Bias in selection of the reported result** | | | |
| --- | --- | --- | --- |
|  | Is the reported effect estimate likely to be selected, on the basis of the results, from... |  |  |
|  | 7.1. ... multiple outcome *measurements* within the outcome domain? | The outcome results are reported using specific measurements that have stated in the methodology | Y / PY / PN / N / NI |
|  | 7.2 ... multiple *analyses* of the intervention-outcome relationship? | There is no missing of data for the outcomes results | N |
|  | 7.3 ... different *subgroups*? | No subgroup | N |
|  | **Risk of bias judgement** |  | Low |
|  | Optional: What is the predicted direction of bias due to selection of the reported result? |  |  |

| **Overall bias** | | | |
| --- | --- | --- | --- |
|  | **Risk of bias judgement** |  | Low |
|  | Optional: What is the overall predicted direction of bias for this outcome? |  |  |

The Risk Of Bias In Non-randomized Studies – of Interventions (ROBINS-I) assessment tool

**Version 19 September 2016**

Article title: Post-exercise ingestion of different amounts of protein affects plasma insulin concentration in humans

Authors: Morifuji, M.; Aoyama, T.; Nakata, A.; Sambongi, C.; Koga, J.; Kurihara, K.; Kanegae, M.; Suzuki, K.; Higuchi, M.


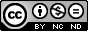


This work is licensed under a [Creative Commons Attribution-NonCommercial-NoDerivatives 4.0 International License](http://creativecommons.org/licenses/by-nc-nd/4.0/).

# ROBINS-I tool (Stage I): At protocol stage

## Specify the review question

| Participants | Athletes experience recovering from injuries and/or hinder in performance. |
| --- | --- |
| Experimental intervention | Whey protein or the whey supplements. It can be in form of isolate, concentrate, or hydrolysate. |
| Comparator | Carbohydrate supplement; protein-containing foods include animal sources, and vegetarian sources; vitamins; minerals; placebo |
| Outcomes | level of the protein in blood and creatine kinase activity; development of muscle and bone tissue; the muscle and bone fracture injuries recovery period; pain level; and mobility, strength and performance level |

## List the confounding domains relevant to all or most studies

| Consume whey protein |
| --- |

## List co-interventions that could be different between intervention groups and that could impact on outcomes

| None |
| --- |

# ROBINS-I tool (Stage II): For each study

## Specify a target randomized trial specific to the study

| Design | Matched (cross-over) |
| --- | --- |
| Participants | Trained men |
| Experimental intervention | (1) carbohydrate plus a low amount of whey protein (2) carbohydrate plus a high amount of whey protein |
| Comparator | carbohydrate |

## Is your aim for this study…?

| ☑ | to assess the effect of *assignment to* intervention |
| --- | --- |
| □ | to assess the effect of *starting and adhering to* intervention |

## Specify the outcome

Specify which outcome is being assessed for risk of bias (typically from among those earmarked for the Summary of Findings table). Specify whether this is a proposed benefit or harm of intervention.

| Glucose, essential amino acids, branched-chain amino acids, insulin |
| --- |

## Specify the numerical result being assessed

In case of multiple alternative analyses being presented, specify the numeric result (e.g. RR = 1.52 (95% CI 0.83 to 2.77) and/or a reference (e.g. to a table, figure or paragraph) that uniquely defines the result being assessed.

| Figure 1. (A) Blood glucose and (B) plasma insulin concentrations; Figure 2. Plasma concentrations of (B) essential amino acids, and (C) branched-chain amino acids; Table I. Characteristics of participants |
| --- |

## Preliminary consideration of confounders

Complete a row for each important confounding domain (i) listed in the review protocol; and (ii) relevant to the setting of this particular study, or which the study authors identified as potentially important.

#### “Important” confounding domains are those for which, in the context of this study, adjustment is expected to lead to a clinically important change in the estimated effect of the intervention. “Validity” refers to whether the confounding variable or variables fully measure the domain, while “reliability” refers to the precision of the measurement (more measurement error means less reliability).

| **(i) Confounding domains listed in the review protocol** | | | | |
| --- | --- | --- | --- | --- |
| Confounding domain | Measured variable(s) | Is there evidence that controlling for this variable was unnecessary?* | Is the confounding domain measured validly and reliably by this variable (or these variables)? | OPTIONAL: Is failure to adjust for this variable (alone) expected to favour the experimental intervention or the comparator? |
| Not Applicable (NA) |  |  | Yes / No / No information | Favour experimental / Favour comparator / No information |
|  |  |  |  |  |
|  |  |  |  |  |
|  |  |  |  |  |
|  |  |  |  |  |
|  |  |  |  |  |
|  |  |  |  |  |
|  |  |  |  |  |
|  |  |  |  |  |

| **(ii) Additional confounding domains relevant to the setting of this particular study, or which the study authors identified as important** | | | | |
| --- | --- | --- | --- | --- |
| Confounding domain | Measured variable(s) | Is there evidence that controlling for this variable was unnecessary?* | Is the confounding domain measured validly and reliably by this variable (or these variables)? | OPTIONAL: Is failure to adjust for this variable (alone) expected to favour the experimental intervention or the comparator? |
| NA |  |  | Yes / No / No information | Favour experimental / Favour comparator / No information |
|  |  |  |  |  |
|  |  |  |  |  |
|  |  |  |  |  |
|  |  |  |  |  |
|  |  |  |  |  |
|  |  |  |  |  |
|  |  |  |  |  |
|  |  |  |  |  |
|  |  |  |  |  |

* In the context of a particular study, variables can be demonstrated not to be confounders and so not included in the analysis: (a) if they are not predictive of the outcome; (b) if they are not predictive of intervention; or (c) because adjustment makes no or minimal difference to the estimated effect of the primary parameter. Note that “no statistically significant association” is not the same as “not predictive”.

## Preliminary consideration of co-interventions

Complete a row for each important co-intervention (i) listed in the review protocol; and (ii) relevant to the setting of this particular study, or which the study authors identified as important.

#### “Important” co-interventions are those for which, in the context of this study, adjustment is expected to lead to a clinically important change in the estimated effect of the intervention.

| **(i) Co-interventions listed in the review protocol** | | |
| --- | --- | --- |
| Co-intervention | Is there evidence that controlling for this co-intervention was unnecessary (e.g. because it was not administered)? | Is presence of this co-intervention likely to favour outcomes in the experimental intervention or the comparator |
| NA |  | Favour experimental / Favour comparator / No information |
|  |  | Favour experimental / Favour comparator / No information |
|  |  | Favour experimental / Favour comparator / No information |
|  |  | Favour experimental / Favour comparator / No information |

| **(ii) Additional co-interventions relevant to the setting of this particular study, or which the study authors identified as important** | | |
| --- | --- | --- |
| Co-intervention | Is there evidence that controlling for this co-intervention was unnecessary (e.g. because it was not administered)? | Is presence of this co-intervention likely to favour outcomes in the experimental intervention or the comparator |
| NA |  | Favour experimental / Favour comparator / No information |
|  |  | Favour experimental / Favour comparator / No information |
|  |  | Favour experimental / Favour comparator / No information |
|  |  | Favour experimental / Favour comparator / No information |

## Risk of bias assessment

Responses underlined in green are potential markers for low risk of bias, and responses in red are potential markers for a risk of bias. Where questions relate only to sign posts to other questions, no formatting is used.

|  | **Signalling questions** | **Description** | **Response options** |
| --- | --- | --- | --- |
| **Bias due to confounding** | | | |
|  | 1.1 Is there potential for confounding of the effect of intervention in this study?  **If N/PN to 1.1:** the study can be considered to be at low risk of bias due to confounding and no further signalling questions need be considered | all participants were instructed to eat the same meals the day before  the test. The calorific intake in the 24-h period before each time trial was 8700 kJ/day. In the hour preceding the tests, the participants were not allowed to eat but were allowed to drink water. | N |
|  | **If Y/PY to 1.1**: determine whether there is a need to assess time-varying confounding: |  |  |
|  | 1.2. Was the analysis based on splitting participants’ follow up time according to intervention received?  **If N/PN**, answer questions relating to baseline confounding (1.4 to 1.6)  **If Y/PY**, go to question 1.3. |  |  |
|  | 1.3. Were intervention discontinuations or switches likely to be related to factors that are prognostic for the outcome?  **If N/PN**, answer questions relating to baseline confounding (1.4 to 1.6)  **If Y/PY**, answer questions relating to both baseline and time-varying confounding (1.7 and 1.8) |  |  |

|  | **Questions relating to baseline confounding only** | | |
| --- | --- | --- | --- |
|  | 1.4. Did the authors use an appropriate analysis method that controlled for all the important confounding domains? |  |  |
|  | 1.5. **If Y/PY to 1.4**: Were confounding domains that were controlled for measured validly and reliably by the variables available in this study? |  |  |
|  | 1.6. Did the authors control for any post-intervention variables that could have been affected by the intervention? |  |  |
|  | **Questions relating to baseline and time-varying confounding** | |  |
|  | 1.7. Did the authors use an appropriate analysis method that controlled for all the important confounding domains and for time-varying confounding? |  |  |
|  | 1.8. **If Y/PY to 1.7**: Were confounding domains that were controlled for measured validly and reliably by the variables available in this study? |  |  |
|  | **Risk of bias judgement** |  | Low |
|  | Optional: What is the predicted direction of bias due to confounding? |  |  |

| **Bias in selection of participants into the study** | | | |
| --- | --- | --- | --- |
|  | 2.1. Was selection of participants into the study (or into the analysis) based on participant characteristics observed after the start of intervention?  **If N/PN to 2.1:** go to 2.4 | The selection of participants into study before starting the protocol of the study. For the protocol and potential benefits and risks associated with participation in the study were explained in full before each participant signed an informed consent document. | N |
|  | 2.2. **If Y/PY to 2.1**: Were the post-intervention variables that influenced selection likely to be associated with intervention?  2.3 **If Y/PY to 2.2**: Were the post-intervention variables that influenced selection likely to be influenced by the outcome or a cause of the outcome? |  |  |
|  | 2.4. Do start of follow-up and start of intervention coincide for most participants? | 8 out of 15 participants. | PY |
|  | 2.5. **If Y/PY to 2.2 and 2.3, or N/PN to 2.4**: Were adjustment techniques used that are likely to correct for the presence of selection biases? |  |  |
|  | **Risk of bias judgement** |  | Low |
|  | Optional: What is the predicted direction of bias due to selection of participants into the study? |  |  |

| **Bias in classification of interventions** | | | |
| --- | --- | --- | --- |
|  | 3.1 Were intervention groups clearly defined? | Table I | Y |
|  | 3.2 Was the information used to define intervention groups recorded at the start of the intervention? | Table I | Y |
|  | 3.3 Could classification of intervention status have been affected by knowledge of the outcome or risk of the outcome? | Collection of the information at the time is sufficient | N |
|  | **Risk of bias judgement** |  | Low |
|  | Optional: What is the predicted direction of bias due to classification of interventions? |  |  |

| **Bias due to deviations from intended interventions** | | | |
| --- | --- | --- | --- |
|  | **If your aim for this study is to assess the effect of assignment to intervention, answer questions 4.1 and 4.2** | |  |
|  | 4.1. Were there deviations from the intended intervention beyond what would be expected in usual practice? | Crossover study. Each participate receive three supplements | N |
|  | 4.2. **If Y/PY to 4.1**: Were these deviations from intended intervention unbalanced between groups *and* likely to have affected the outcome? |  |  |
|  | **If your aim for this study is to assess the effect of starting and adhering to intervention, answer questions 4.3 to 4.6** | |  |
|  | 4.3. Were important co-interventions balanced across intervention groups? |  |  |
|  | 4.4. Was the intervention implemented successfully for most participants? |  |  |
|  | 4.5. Did study participants adhere to the assigned intervention regimen? |  |  |
|  | 4.6. **If N/PN to 4.3, 4.4 or 4.5**: Was an appropriate analysis used to estimate the effect of starting and adhering to the intervention? |  |  |
|  | **Risk of bias judgement** |  | Low |
|  | Optional: What is the predicted direction of bias due to deviations from the intended interventions? |  |  |

| **Bias due to missing data** | | | |
| --- | --- | --- | --- |
|  | 5.1 Were outcome data available for all, or nearly all, participants? | The results based on 8 participants | Y |
|  | 5.2 Were participants excluded due to missing data on intervention status? | Seven participants were subsequently excluded as they were unable to complete the exercise protocol. | PN |
|  | 5.3 Were participants excluded due to missing data on other variables needed for the analysis? | The results based on 8 participants | N |
|  | 5.4 **If PN/N to 5.1, or Y/PY to 5.2 or 5.3**: Are the proportion of participants and reasons for missing data similar across interventions? |  |  |
|  | 5.5 **If PN/N to 5.1, or Y/PY to 5.2 or 5.3**: Is there evidence that results were robust to the presence of missing data? |  |  |
|  | **Risk of bias judgement** |  | Low |
|  | Optional: What is the predicted direction of bias due to missing data? |  |  |

| **Bias in measurement of outcomes** | | | |
| --- | --- | --- | --- |
|  | 6.1 Could the outcome measure have been influenced by knowledge of the intervention received? | The study was conducted according to the guidelines of the Declaration  of Helsinki and all procedures received approval from the Ethics Committee of the Faculty of Sport Sciences, Waseda University. | N |
|  | 6.2 Were outcome assessors aware of the intervention received by study participants? | It is double blinded study design | N |
|  | 6.3 Were the methods of outcome assessment comparable across intervention groups? | Both group outcomes are using the same assessment | Y |
|  | 6.4 Were any systematic errors in measurement of the outcome related to intervention received? | The method impose are well explain for conducting the study and blinded | N |
|  | **Risk of bias judgement** |  | Low |
|  | Optional: What is the predicted direction of bias due to measurement of outcomes? |  |  |

| **Bias in selection of the reported result** | | | |
| --- | --- | --- | --- |
|  | Is the reported effect estimate likely to be selected, on the basis of the results, from... |  |  |
|  | 7.1. ... multiple outcome *measurements* within the outcome domain? | The outcome results are reported using specific measurements that have stated in the section material and method | N |
|  | 7.2 ... multiple *analyses* of the intervention-outcome relationship? | There is no missing of data for the outcomes results upon the 8 participants | N |
|  | 7.3 ... different *subgroups*? | No subgroups | N |
|  | **Risk of bias judgement** |  | Low |
|  | Optional: What is the predicted direction of bias due to selection of the reported result? |  |  |

| **Overall bias** | | | |
| --- | --- | --- | --- |
|  | **Risk of bias judgement** |  | Low |
|  | Optional: What is the overall predicted direction of bias for this outcome? |  |  |


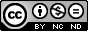


This work is licensed under a [Creative Commons Attribution-NonCommercial-NoDerivatives 4.0 International License](http://creativecommons.org/licenses/by-nc-nd/4.0/).


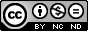


This work is licensed under a [Creative Commons Attribution-NonCommercial-NoDerivatives 4.0 International License](http://creativecommons.org/licenses/by-nc-nd/4.0/).
