# Supplementary material for: Effectiveness of whey protein supplements on the serum levels of amino acid, creatinine kinase and myoglobin of athletes: a systematic review and meta-analysis
Source: Syst Rev. 2019 May 31;8:130. doi: 10.1186/s13643-019-1039-z (PMC6544960; doi:10.1186/s13643-019-1039-z)
Supplement: Supplementary file 3 — Meta-analysis output. (ZIP 162 kb) [file 13643_2019_1039_MOESM3_ESM.zip › supplementary materialTable 3 20181115R1.docx]

Supplementary material Table 3: Meta-analysis output

#### Essential amino acid

##### Forest Plot

Study | WMD [95% Conf. Interval] % Weight

---------------------+---------------------------------------------------

Areta -A (2014) |700.000 295.120 1104.880 15.30

Areta -B (2014) |1000.000 569.821 1430.179 15.06

Impey -A (2015) | 50.000 37.684 62.316 17.41

Impey -B (2015) |1202.000 1192.699 1211.301 17.41

Parr (2014) |402.000 386.505 417.495 17.41

Tang (2007) |450.000 437.750 462.250 17.41

---------------------+---------------------------------------------------

D+L pooled WMD |624.035 169.270 1078.799 100.00

---------------------+---------------------------------------------------

Heterogeneity chi-squared = 24656.96 (d.f. = 5) p = 0.000

I-squared (variation in WMD attributable to heterogeneity) = 100.0%

Estimate of between-study variance Tau-squared = 3.1e+05

Test of WMD=0 : z= 2.69 p = 0.007

Areta -A (2014) = 15 g WP vs placebo

Areta -B (2014) = 30 g WP vs placebo

Impey -A (2015) = WP vs carbohydrate

Impey -B (2015) = WP with caffeine vs carbohydrate

##### Subgroup

###### Subgroup by physical activities

Study | WMD [95% Conf. Interval]

---------------------+---------------------------------------------------

leg

Areta -A (2014) | 700.000 295.120 1104.880

Areta -B (2014) | 1000.000 569.821 1430.179

Parr (2014) | 402.000 386.505 417.495

Sub-total |

D+L pooled WMD | 653.764 277.576 1029.952

---------------------+---------------------------------------------------

cycle

Impey -A (2015) | 50.000 37.684 62.316

Impey -B (2015) | 1202.000 1192.699 1211.301

Sub-total |

D+L pooled WMD | 626.007 -502.932 1754.947

---------------------+---------------------------------------------------

gym

Tang (2007) | 450.000 437.750 462.250

Sub-total |

D+L pooled WMD | 450.000 437.750 462.250

---------------------+---------------------------------------------------

Test(s) of heterogeneity:

Heterogeneity degrees of

statistic freedom P I-squared** Tau-squared

leg 9.48 2 0.009 78.9% 8.5e+04

cycle 21402.34 1 0.000 100.0% 6.6e+05

gym 0.00 0 . .% 0.0000

** I-squared: the variation in WMD attributable to heterogeneity)

Significance test(s) of WMD=0

leg z= 3.41 p = 0.001

cycle z= 1.09 p = 0.277

gym z= 72.00 p = 0.000

Areta -A (2014) = 15 g WP vs placebo

Areta -B (2014) = 30 g WP vs placebo

Impey -A (2015) = WP vs carbohydrate

Impey -B (2015) = WP with caffeine vs carbohydrate

###### Subgroup by intervention period range (day)

Study | WMD [95% Conf. Interval]

---------------------+---------------------------------------------------

41-60

Areta -A (2014) | 700.000 295.120 1104.880

Areta -B (2014) | 1000.000 569.821 1430.179

Sub-total |

D+L pooled WMD | 840.919 546.088 1135.751

---------------------+---------------------------------------------------

1-20

Impey -A (2015) | 50.000 37.684 62.316

Impey -B (2015) | 1202.000 1192.699 1211.301

Parr (2014) | 402.000 386.505 417.495

Tang (2007) | 450.000 437.750 462.250

Sub-total |

D+L pooled WMD | 526.012 -19.143 1071.167

---------------------+---------------------------------------------------

Test(s) of heterogeneity:

Heterogeneity degrees of

statistic freedom P I-squared** Tau-squared

41-60 0.99 1 0.320 0.0% 0.0000

1-20 24654.40 3 0.000 100.0% 3.1e+05

** I-squared: the variation in WMD attributable to heterogeneity)

Significance test(s) of WMD=0

41-60 z= 5.59 p = 0.000

1-20 z= 1.89 p = 0.059

Areta -A (2014) = 15 g WP vs placebo

Areta -B (2014) = 30 g WP vs placebo

Impey -A (2015) = WP vs carbohydrate

Impey -B (2015) = WP with caffeine vs carbohydrate

#### Branched-chain amino acid

##### Forest Plot

Study | WMD [95% Conf. Interval] % Weight

---------------------+---------------------------------------------------

Areta -A (2014) |310.000 274.216 345.784 11.14

Areta -B (2014) |610.000 533.922 686.078 11.07

Impey -A (2015) | 50.000 45.380 54.620 11.16

Impey -B (2015) |660.000 655.380 664.620 11.16

Morifuji -A (2012) |500.000 497.535 502.465 11.16

Morifuji -B (2012) |1300.000 1296.306 1303.694 11.16

Parr -A (2014) |110.000 12.002 207.998 11.01

Parr -B (2014) |210.000 112.002 307.998 11.01

Tang (2007) |370.000 366.531 373.469 11.16

---------------------+---------------------------------------------------

D+L pooled WMD |458.572 179.959 737.184 100.00

---------------------+---------------------------------------------------

Heterogeneity chi-squared = 2.2e+05 (d.f. = 8) p = 0.000

I-squared (variation in WMD attributable to heterogeneity) = 100.0%

Estimate of between-study variance Tau-squared = 1.8e+05

Test of WMD=0 : z= 3.23 p = 0.001

Areta -A (2014) = 15 g WP vs placebo

Areta -B (2014) = 30 g WP vs placebo

Impey -A (2015) = WP vs carbohydrate

Impey -B (2015) = WP with caffeine vs carbohydrate

Morifuji -A (2012) = 3.0 g WP vs carbohydrate

Morifuji -B (2012) = 8.0 g WP vs carbohydrate

Parr -A (2014) = 25 g WP vs maltodextrin with alcohol

Parr -B (2014) = 25 g WP with alcohol vs maltodextrin with alcohol

##### Subgroup

###### Subgroup by physical activities

Study | WMD [95% Conf. Interval]

---------------------+---------------------------------------------------

leg

Areta -A (2014) | 310.000 274.216 345.784

Areta -B (2014) | 610.000 533.922 686.078

Parr -A (2014) | 110.000 12.002 207.998

Parr -B (2014) | 210.000 112.002 307.998

Sub-total |

D+L pooled WMD | 312.105 129.371 494.838

---------------------+---------------------------------------------------

cycle

Impey -A (2015) | 50.000 45.380 54.620

Impey -B (2015) | 660.000 655.380 664.620

Morifuji -A (2012) | 500.000 497.535 502.465

Morifuji -B (2012) | 1300.000 1296.306 1303.694

Sub-total |

D+L pooled WMD | 627.501 153.985 1101.016

---------------------+---------------------------------------------------

gym

Tang (2007) | 370.000 366.531 373.469

Sub-total |

D+L pooled WMD | 370.000 366.531 373.469

---------------------+---------------------------------------------------

Test(s) of heterogeneity:

Heterogeneity degrees of

statistic freedom P I-squared** Tau-squared

leg 78.33 3 0.000 96.2% 3.3e+04

cycle 2.0e+05 3 0.000 100.0% 2.3e+05

gym 0.00 0 . .% 0.0000

** I-squared: the variation in WMD attributable to heterogeneity)

Significance test(s) of WMD=0

leg z= 3.35 p = 0.001

cycle z= 2.60 p = 0.009

gym z= 209.04 p = 0.000

Areta -A (2014) = 15 g WP vs placebo

Areta -B (2014) = 30 g WP vs placebo

Impey -A (2015) = WP vs carbohydrate

Impey -B (2015) = WP with caffeine vs carbohydrate

Morifuji -A (2012) = 3.0 g WP vs carbohydrate

Morifuji -B (2012) = 8.0 g WP vs carbohydrate

Parr -A (2014) = 25 g WP vs maltodextrin with alcohol

Parr -B (2014) = 25 g WP with alcohol vs maltodextrin with alcohol

###### Subgroup by intervention period range (day)

Study | WMD [95% Conf. Interval]

---------------------+---------------------------------------------------

41-60

Areta -A (2014) | 310.000 274.216 345.784

Areta -B (2014) | 610.000 533.922 686.078

Sub-total |

D+L pooled WMD | 458.044 164.075 752.014

---------------------+---------------------------------------------------

1-20

Impey -A (2015) | 50.000 45.380 54.620

Impey -B (2015) | 660.000 655.380 664.620

Morifuji -A (2012) | 500.000 497.535 502.465

Morifuji -B (2012) | 1300.000 1296.306 1303.694

Parr -A (2014) | 110.000 12.002 207.998

Parr -B (2014) | 210.000 112.002 307.998

Tang (2007) | 370.000 366.531 373.469

Sub-total |

D+L pooled WMD | 458.299 141.976 774.621

---------------------+---------------------------------------------------

Test(s) of heterogeneity:

Heterogeneity degrees of

statistic freedom P I-squared** Tau-squared

41-60 48.91 1 0.000 98.0% 4.4e+04

1-20 2.2e+05 6 0.000 100.0% 1.8e+05

** I-squared: the variation in WMD attributable to heterogeneity)

Significance test(s) of WMD=0

41-60 z= 3.05 p = 0.002

1-20 z= 2.84 p = 0.005

Areta -A (2014) = 15 g WP vs placebo

Areta -B (2014) = 30 g WP vs placebo

Impey -A (2015) = WP vs carbohydrate

Impey -B (2015) = WP with caffeine vs carbohydrate

Morifuji -A (2012) = 3.0 g WP vs carbohydrate

Morifuji -B (2012) = 8.0 g WP vs carbohydrate

Parr -A (2014) = 25 g WP vs maltodextrin with alcohol

Parr -B (2014) = 25 g WP with alcohol vs maltodextrin with alcohol

#### Creatine kinase

##### Forest Plot

Study | WMD [95% Conf. Interval] % Weight

---------------------+---------------------------------------------------

Cepero -A (2010) | 23.800 -27.162 74.762 8.13

Cepero -B (2010) | 15.400 -32.011 62.811 8.16

Gunnarsson (2013) | 71.000 42.506 99.494 8.30

Hansen (2015) |-430.000 -462.669 -397.331 8.28

Hansen (2016) | 0.000 -10.782 10.782 8.38

Jauhari -A (2014) | 22.000 -22.037 66.037 8.19

Jauhari -B (2014) |-77.000 -235.270 81.270 6.41

Kraemer (2015) |100.000 65.620 134.380 8.27

Lollo -A (2014) |-112.370 -162.671 -62.069 8.13

Lollo -B (2014) |-164.790 -216.355 -113.225 8.12

Naclerio -A (2015) | 83.500 -89.038 256.038 6.13

Naclerio -B (2015) |-37.300 -258.006 183.406 5.24

Yang (2014) |-76.470 -110.701 -42.239 8.27

---------------------+---------------------------------------------------

D+L pooled WMD |-47.049 -129.465 35.367 100.00

---------------------+---------------------------------------------------

Heterogeneity chi-squared = 766.54 (d.f. = 12) p = 0.000

I-squared (variation in WMD attributable to heterogeneity) = 98.4%

Estimate of between-study variance Tau-squared = 2.1e+04

Test of WMD=0 : z= 1.12 p = 0.263

Cepero -A (2010) = WP vs carbohydrate

Cepero -B (2010) = WP vs casein

Jauhari -A (2014) = WP vs tempeh

Jauhari -B (2014) = WP vs placebo

Lollo -A (2014) = WP concentrate vs maltodextrin

Lollo -B (2014) = WP hydrolysed vs maltodextrin

Naclerio -A (2015) = WP with multi-ingredient vs carbohydrate

Naclerio -B (2015) = WP with multi-ingredient vs placebo

##### Funnel Plot

##### Egger test

Egger test for small-study effects:

Regress standard normal deviate of intervention

effect estimate against its standard error

Number of studies = 13 Root MSE = 8.219

------------------------------------------------------------------------------

Std_Eff | Coef. Std. Err. t P>|t| [95% Conf. Interval]

-------------+----------------------------------------------------------------

slope | -1.055819 54.17511 -0.02 0.985 -120.2944 118.1828

bias | -2.103328 3.568176 -0.59 0.567 -9.956831 5.750175

------------------------------------------------------------------------------

Test of H0: no small-study effects P = 0.567

##### Subgroup

###### Subgroup by physical activities

Study | WMD [95% Conf. Interval]

---------------------+---------------------------------------------------

cycle

Cepero -A (2010) | 23.800 -27.162 74.762

Cepero -B (2010) | 15.400 -32.011 62.811

Sub-total |

D+L pooled WMD | 19.297 -15.415 54.009

---------------------+---------------------------------------------------

soccer

Gunnarsson (2013) | 71.000 42.506 99.494

Lollo -A (2014) | -112.370 -162.671 -62.069

Lollo -B (2014) | -164.790 -216.355 -113.225

Sub-total |

D+L pooled WMD | -67.582 -225.190 90.026

---------------------+---------------------------------------------------

run

Hansen (2015) | -430.000 -462.669 -397.331

Naclerio -A (2015) | 83.500 -89.038 256.038

Naclerio -B (2015) | -37.300 -258.006 183.406

Yang (2014) | -76.470 -110.701 -42.239

Sub-total |

D+L pooled WMD | -124.302 -376.917 128.314

---------------------+---------------------------------------------------

cycle and resistance

Hansen (2016) | 0.000 -10.782 10.782

Kraemer (2015) | 100.000 65.620 134.380

Sub-total |

D+L pooled WMD | 48.613 -49.348 146.573

---------------------+---------------------------------------------------

resistance exercise

Jauhari -A (2014) | 22.000 -22.037 66.037

Jauhari -B (2014) | -77.000 -235.270 81.270

Sub-total |

D+L pooled WMD | 2.884 -73.707 79.475

---------------------+---------------------------------------------------

Test(s) of heterogeneity:

Heterogeneity degrees of

statistic freedom P I-squared** Tau-squared

cycle 0.06 1 0.813 0.0% 0.0000

soccer 81.57 2 0.000 97.5% 1.9e+04

run 233.21 3 0.000 98.7% 6.2e+04

cycle and resistance 29.59 1 0.000 96.6% 4.8e+03

resistance exercise 1.40 1 0.238 28.3% 1.4e+03

** I-squared: the variation in WMD attributable to heterogeneity)

Significance test(s) of WMD=0

cycle z= 1.09 p = 0.276

soccer z= 0.84 p = 0.401

run z= 0.96 p = 0.335

cycle and resistance z= 0.97 p = 0.331

resistance exercise z= 0.07 p = 0.941

Cepero -A (2010) = WP vs carbohydrate

Cepero -B (2010) = WP vs casein

Jauhari -A (2014) = WP vs tempeh

Jauhari -B (2014) = WP vs placebo

Lollo -A (2014) = WP concentrate vs maltodextrin

Lollo -B (2014) = WP hydrolysed vs maltodextrin

Naclerio -A (2015) = WP with multi-ingredient vs carbohydrate

Naclerio -B (2015) = WP with multi-ingredient vs placebo

###### Subgroup by intervention period range (day)

Study | WMD [95% Conf. Interval]

---------------------+---------------------------------------------------

1-20

Cepero -A (2010) | 23.800 -27.162 74.762

Cepero -B (2010) | 15.400 -32.011 62.811

Gunnarsson (2013) | 71.000 42.506 99.494

Hansen (2015) | -430.000 -462.669 -397.331

Hansen (2016) | 0.000 -10.782 10.782

Jauhari -A (2014) | 22.000 -22.037 66.037

Jauhari -B (2014) | -77.000 -235.270 81.270

Naclerio -A (2015) | 83.500 -89.038 256.038

Naclerio -B (2015) | -37.300 -258.006 183.406

Yang (2014) | -76.470 -110.701 -42.239

Sub-total |

D+L pooled WMD | -43.120 -144.598 58.358

---------------------+---------------------------------------------------

41-60

Kraemer (2015) | 100.000 65.620 134.380

Sub-total |

D+L pooled WMD | 100.000 65.620 134.380

---------------------+---------------------------------------------------

161-180

Lollo -A (2014) | -112.370 -162.671 -62.069

Lollo -B (2014) | -164.790 -216.355 -113.225

Sub-total |

D+L pooled WMD | -138.260 -189.627 -86.893

---------------------+---------------------------------------------------

Test(s) of heterogeneity:

Heterogeneity degrees of

statistic freedom P I-squared** Tau-squared

1-20 675.73 9 0.000 98.7% 2.4e+04

41-60 0.00 0 . .% 0.0000

161-180 2.03 1 0.154 50.8% 698.5175

** I-squared: the variation in WMD attributable to heterogeneity)

Significance test(s) of WMD=0

1-20 z= 0.83 p = 0.405

41-60 z= 5.70 p = 0.000

161-180 z= 5.28 p = 0.000

Cepero -A (2010) = WP vs carbohydrate

Cepero -B (2010) = WP vs casein

Jauhari -A (2014) = WP vs tempeh

Jauhari -B (2014) = WP vs placebo

Lollo -A (2014) = WP concentrate vs maltodextrin

Lollo -B (2014) = WP hydrolysed vs maltodextrin

Naclerio -A (2015) = WP with multi-ingredient vs carbohydrate

Naclerio -B (2015) = WP with multi-ingredient vs placebo

#### Myoglobin

##### Forest Plot

Study | WMD [95% Conf. Interval] % Weight

---------------------+---------------------------------------------------

Gunnarsson (2013) | 0.000 -11.335 11.335 40.95

Naclerio -A (2015) | -4.200 -12.571 4.171 44.02

Naclerio -B (2015) |-65.800 -105.429 -26.171 15.03

---------------------+---------------------------------------------------

D+L pooled WMD |-11.737 -30.239 6.765 100.00

---------------------+---------------------------------------------------

Heterogeneity chi-squared = 9.80 (d.f. = 2) p = 0.007

I-squared (variation in WMD attributable to heterogeneity) = 79.6%

Estimate of between-study variance Tau-squared = 184.1681

Test of WMD=0 : z= 1.24 p = 0.214

Naclerio -A (2015) = WP with multi-ingredient vs carbohydrate

Naclerio -B (2015) = WP with multi-ingredient vs placebo

##### Subgroup

###### Subgroup by physical activities

Study | WMD [95% Conf. Interval]

---------------------+---------------------------------------------------

soccer game

Gunnarsson (2013) | 0.000 -11.335 11.335

Sub-total |

D+L pooled WMD | 0.000 -11.335 11.335

---------------------+---------------------------------------------------

run, jogging

Naclerio -A (2015) | -4.200 -12.571 4.171

Naclerio -B (2015) | -65.800 -105.429 -26.171

Sub-total |

D+L pooled WMD | -31.830 -91.876 28.217

---------------------+---------------------------------------------------

Test(s) of heterogeneity:

Heterogeneity degrees of

statistic freedom P I-squared** Tau-squared

soccer game 0.00 0 . .% 0.0000

run, jogging 8.89 1 0.003 88.7% 1.7e+03

** I-squared: the variation in WMD attributable to heterogeneity)

Significance test(s) of WMD=0

soccer game z= 0.00 p = 1.000

run, jogging z= 1.04 p = 0.299

Naclerio -A (2015) = WP with multi-ingredient vs carbohydrate

Naclerio -B (2015) = WP with multi-ingredient vs placebo

###### Subgroup by intervention period range (day)

Study | WMD [95% Conf. Interval]

---------------------+---------------------------------------------------

1-20

Gunnarsson (2013) | 0.000 -11.335 11.335

Naclerio -A (2015) | -4.200 -12.571 4.171

Naclerio -B (2015) | -65.800 -105.429 -26.171

Sub-total |

D+L pooled WMD | -11.737 -30.239 6.765

---------------------+---------------------------------------------------

Test(s) of heterogeneity:

Heterogeneity degrees of

statistic freedom P I-squared** Tau-squared

1-20 9.80 2 0.007 79.6% 184.1681

** I-squared: the variation in WMD attributable to heterogeneity)

Significance test(s) of WMD=0

1-20 z= 1.24 p = 0.214

Naclerio -A (2015) = WP with multi-ingredient vs carbohydrate

Naclerio -B (2015) = WP with multi-ingredient vs placebo
