# Supplementary material for: Effectiveness of whey protein supplements on the serum levels of amino acid, creatinine kinase and myoglobin of athletes: a systematic review and meta-analysis
Source: Syst Rev. 2019 May 31;8:130. doi: 10.1186/s13643-019-1039-z (PMC6544960; doi:10.1186/s13643-019-1039-z)
Supplement: Supplementary file 3 — Meta-analysis output. (ZIP 162 kb) [file 13643_2019_1039_MOESM3_ESM.zip › supplementary materialTable 3 Meta analysis outputR1.docx]

# Analysis 1: Essential Amino Acid (nmol/l)

## Random effect

Study | WMD [95% Conf. Interval] % Weight

---------------------+---------------------------------------------------

Areta (2014) |700.000 295.120 1104.880 15.30

Areta (2014) |1000.000 569.821 1430.179 15.06

Impey (2015) | 50.000 37.684 62.316 17.41

Impey (2015) |1202.000 1192.699 1211.301 17.41

Parr (2014) |402.000 386.505 417.495 17.41

Tang (2007) |450.000 437.750 462.250 17.41

---------------------+---------------------------------------------------

D+L pooled WMD |624.035 169.270 1078.799 100.00

---------------------+---------------------------------------------------

Heterogeneity chi-squared = 24656.96 (d.f. = 5) p = 0.000

I-squared (variation in WMD attributable to heterogeneity) = 100.0%

Estimate of between-study variance Tau-squared = 3.1e+05

Test of WMD=0 : z= 2.69 p = 0.007

Figure 1 Meta-Analysis Output on EAA (nmol/l)

# Analysis 2: Branched-chain Amino Acids (nmol/ml)

## Random effect

Study | WMD [95% Conf. Interval] % Weight

---------------------+---------------------------------------------------

Areta (2014) |310.000 274.216 345.784 11.14

Areta (2014) |610.000 533.922 686.078 11.07

Impey (2015) | 50.000 45.380 54.620 11.16

Impey (2015) |660.000 655.380 664.620 11.16

Morifuji (2012) |500.000 497.535 502.465 11.16

Morifuji (2012) |1300.000 1296.306 1303.694 11.16

Parr (2014) |110.000 12.002 207.998 11.01

Parr (2014) |210.000 112.002 307.998 11.01

Tang (2007) |370.000 366.531 373.469 11.16

---------------------+---------------------------------------------------

D+L pooled WMD |458.572 179.959 737.184 100.00

---------------------+---------------------------------------------------

Heterogeneity chi-squared = 2.2e+05 (d.f. = 8) p = 0.000

I-squared (variation in WMD attributable to heterogeneity) = 100.0%

Estimate of between-study variance Tau-squared = 1.8e+05

Test of WMD=0 : z= 3.23 p = 0.001

Figure 2 Meta-Analysis Output on BCAA (nmol/ml)

# Analysis 3: Myoglobin (ng/ml)

## Random effect

Study | WMD [95% Conf. Interval] % Weight

---------------------+---------------------------------------------------

Gunnarsson (2013) | 0.000 -11.335 11.335 40.95

Naclerio (2015) | -4.200 -12.571 4.171 44.02

Naclerio (2015) |-65.800 -105.429 -26.171 15.03

---------------------+---------------------------------------------------

D+L pooled WMD |-11.737 -30.239 6.765 100.00

---------------------+---------------------------------------------------

Heterogeneity chi-squared = 9.80 (d.f. = 2) p = 0.007

I-squared (variation in WMD attributable to heterogeneity) = 79.6%

Estimate of between-study variance Tau-squared = 184.1681

Test of WMD=0 : z= 1.24 p = 0.214

Figure 3 Meta-Analysis Output on Myoglobin (ng/ml)

# Analysis 4: Cortisol (nmol/l)

## Random effect

Study | WMD [95% Conf. Interval] % Weight

---------------------+---------------------------------------------------

Hansen (2015) | 40.000 9.779 70.221 2.29

Hansen (2016) |-15.000 -28.643 -1.357 8.87

Kraemer (2015) | 40.000 -148.640 228.640 0.06

Mero (1997) | -1.000 -4.078 2.078 29.25

Mero (1997) | -9.000 -12.926 -5.074 27.15

Nelson (2013) |-26.000 -225.930 173.930 0.06

Shing (2013) | -7.000 -8.479 -5.521 32.32

---------------------+---------------------------------------------------

D+L pooled WMD | -5.401 -10.143 -0.659 100.00

---------------------+---------------------------------------------------

Heterogeneity chi-squared = 24.88 (d.f. = 6) p = 0.000

I-squared (variation in WMD attributable to heterogeneity) = 75.9%

Estimate of between-study variance Tau-squared = 17.5409

Test of WMD=0 : z= 2.23 p = 0.026

Figure 4 Meta-Analysis Output on Cortisol (nmol/l)

## Subgroup

### by group similarity exercise activity during study

Study | WMD [95% Conf. Interval]

---------------------+---------------------------------------------------

run

Hansen (2015) | 40.000 9.779 70.221

Sub-total |

D+L pooled WMD | 40.000 9.779 70.221

---------------------+---------------------------------------------------

cycle

Hansen (2016) | -15.000 -28.643 -1.357

Kraemer (2015) | 40.000 -148.640 228.640

Nelson (2013) | -26.000 -225.930 173.930

Shing (2013) | -7.000 -8.479 -5.521

Sub-total |

D+L pooled WMD | -7.091 -8.561 -5.621

---------------------+---------------------------------------------------

leg

Mero (1997) | -1.000 -4.078 2.078

Mero (1997) | -9.000 -12.926 -5.074

Sub-total |

D+L pooled WMD | -4.903 -12.741 2.934

---------------------+---------------------------------------------------

Test(s) of heterogeneity:

Heterogeneity degrees of

statistic freedom P I-squared** Tau-squared

run 0.00 0 . .% 0.0000

cycle 1.58 3 0.664 0.0% 0.0000

leg 9.88 1 0.002 89.9% 28.7605

** I-squared: the variation in WMD attributable to heterogeneity)

Significance test(s) of WMD=0

run z= 2.59 p = 0.009

cycle z= 9.45 p = 0.000

leg z= 1.23 p = 0.220

Figure 5 Subgroup Meta-Analysis Output on Cortisol (nmol/l) by Grouping Similarity Exercise

Figure 6 Forest Plot of Subgroup Meta-Analysis on Cortisol (nmol/l) by Grouping Similarity Exercise

### by duration of the studies period (day)

Study | WMD [95% Conf. Interval]

---------------------+---------------------------------------------------

7

Hansen (2015) | 40.000 9.779 70.221

Hansen (2016) | -15.000 -28.643 -1.357

Sub-total |

D+L pooled WMD | 10.779 -43.014 64.573

---------------------+---------------------------------------------------

56

Kraemer (2015) | 40.000 -148.640 228.640

Shing (2013) | -7.000 -8.479 -5.521

Sub-total |

D+L pooled WMD | -6.997 -8.476 -5.518

---------------------+---------------------------------------------------

41

Mero (1997) | -1.000 -4.078 2.078

Mero (1997) | -9.000 -12.926 -5.074

Sub-total |

D+L pooled WMD | -4.903 -12.741 2.934

---------------------+---------------------------------------------------

26

Nelson (2013) | -26.000 -225.930 173.930

Sub-total |

D+L pooled WMD | -26.000 -225.930 173.930

---------------------+---------------------------------------------------

Test(s) of heterogeneity:

Heterogeneity degrees of

statistic freedom P I-squared** Tau-squared

7 10.57 1 0.001 90.5% 1.4e+03

56 0.24 1 0.625 0.0% 0.0000

41 9.88 1 0.002 89.9% 28.7605

26 0.00 0 . .% 0.0000

** I-squared: the variation in WMD attributable to heterogeneity)

Significance test(s) of WMD=0

7 z= 0.39 p = 0.695

56 z= 9.27 p = 0.000

41 z= 1.23 p = 0.220

26 z= 0.25 p = 0.799

Figure 7 Subgroup Meta-Analysis Output on Cortisol (nmol/l) by Duration of the Studies Period

Figure 8 Forest Plot of Subgroup Meta-Analysis on Cortisol (nmol/l)) by Duration of the Studies Period

# Analysis 5: Creatine Kinase (u/l)

## Random effect

Study | WMD [95% Conf. Interval] % Weight

---------------------+---------------------------------------------------

Cepero (2010) | 23.800 -27.162 74.762 8.13

Cepero (2010) | 15.400 -32.011 62.811 8.16

Gunnarsson (2013) | 71.000 42.506 99.494 8.30

Hansen (2015) |-430.000 -462.669 -397.331 8.28

Hansen (2016) | 0.000 -10.782 10.782 8.38

Jauhari (2014) | 22.000 -22.037 66.037 8.19

Jauhari (2014) |-77.000 -235.270 81.270 6.41

Kraemer (2015) |100.000 65.620 134.380 8.27

Lollo (2014) |-112.370 -162.671 -62.069 8.13

Lollo (2014) |-164.790 -216.355 -113.225 8.12

Naclerio (2015) | 83.500 -89.038 256.038 6.13

Naclerio (2015) |-37.300 -258.006 183.406 5.24

Yang (2014) |-76.470 -110.701 -42.239 8.27

---------------------+---------------------------------------------------

D+L pooled WMD |-47.049 -129.465 35.367 100.00

---------------------+---------------------------------------------------

Heterogeneity chi-squared = 766.54 (d.f. = 12) p = 0.000

I-squared (variation in WMD attributable to heterogeneity) = 98.4%

Estimate of between-study variance Tau-squared = 2.1e+04

Test of WMD=0 : z= 1.12 p = 0.263

Figure 9 Meta-Analysis Output on Creatine Kinase (u/l)

## Funnel Plot

Figure 10 Funnel Plot of Creatine Kinase (u/l)

### Egger's test

Egger's test for small-study effects:

Regress standard normal deviate of intervention

effect estimate against its standard error

Number of studies = 13 Root MSE = 8.219

------------------------------------------------------------------------------

Std_Eff | Coef. Std. Err. t P>|t| [95% Conf. Interval]

-------------+----------------------------------------------------------------

slope | -1.055819 54.17511 -0.02 0.985 -120.2944 118.1828

bias | -2.103328 3.568176 -0.59 0.567 -9.956831 5.750175

------------------------------------------------------------------------------

Test of H0: no small-study effects P = 0.567

Figure 11 Egger Test Output of Creatine Kinase (u/l)

Figure 12 Funnel Plot of Egger test on Creatine Kinase (u/l)

## Subgroup

### by group similarity exercise activity during study

Study | WMD [95% Conf. Interval]

---------------------+---------------------------------------------------

cycle

Cepero (2010) | 23.800 -27.162 74.762

Cepero (2010) | 15.400 -32.011 62.811

Sub-total |

D+L pooled WMD | 19.297 -15.415 54.009

---------------------+---------------------------------------------------

soccer

Gunnarsson (2013) | 71.000 42.506 99.494

Lollo (2014) | -112.370 -162.671 -62.069

Lollo (2014) | -164.790 -216.355 -113.225

Sub-total |

D+L pooled WMD | -67.582 -225.190 90.026

---------------------+---------------------------------------------------

run

Hansen (2015) | -430.000 -462.669 -397.331

Naclerio (2015) | 83.500 -89.038 256.038

Naclerio (2015) | -37.300 -258.006 183.406

Yang (2014) | -76.470 -110.701 -42.239

Sub-total |

D+L pooled WMD | -124.302 -376.917 128.314

---------------------+---------------------------------------------------

cycle and resistance

Hansen (2016) | 0.000 -10.782 10.782

Kraemer (2015) | 100.000 65.620 134.380

Sub-total |

D+L pooled WMD | 48.613 -49.348 146.573

---------------------+---------------------------------------------------

resistance exercise

Jauhari (2014) | 22.000 -22.037 66.037

Jauhari (2014) | -77.000 -235.270 81.270

Sub-total |

D+L pooled WMD | 2.884 -73.707 79.475

---------------------+---------------------------------------------------

Test(s) of heterogeneity:

Heterogeneity degrees of

statistic freedom P I-squared** Tau-squared

cycle 0.06 1 0.813 0.0% 0.0000

soccer 81.57 2 0.000 97.5% 1.9e+04

run 233.21 3 0.000 98.7% 6.2e+04

cycle and resistance 29.59 1 0.000 96.6% 4.8e+03

resistance exercise 1.40 1 0.238 28.3% 1.4e+03

** I-squared: the variation in WMD attributable to heterogeneity)

Significance test(s) of WMD=0

cycle z= 1.09 p = 0.276

soccer z= 0.84 p = 0.401

run z= 0.96 p = 0.335

cycle and resistance z= 0.97 p = 0.331

resistance exercise z= 0.07 p = 0.941

Figure 13 Subgroup Meta-Analysis Output on Creatine Kinase (u/l) by Grouping Similarity Exercise

Figure 14 Forest Plot of Subgroup Meta-Analysis on Creatine Kinase (u/l) by Grouping Similarity Exercise

### by range duration of the studies period (day)

Study | WMD [95% Conf. Interval]

---------------------+---------------------------------------------------

1-20

Cepero (2010) | 23.800 -27.162 74.762

Cepero (2010) | 15.400 -32.011 62.811

Gunnarsson (2013) | 71.000 42.506 99.494

Hansen (2015) | -430.000 -462.669 -397.331

Hansen (2016) | 0.000 -10.782 10.782

Jauhari (2014) | 22.000 -22.037 66.037

Jauhari (2014) | -77.000 -235.270 81.270

Naclerio (2015) | 83.500 -89.038 256.038

Naclerio (2015) | -37.300 -258.006 183.406

Yang (2014) | -76.470 -110.701 -42.239

Sub-total |

D+L pooled WMD | -43.120 -144.598 58.358

---------------------+---------------------------------------------------

41-60

Kraemer (2015) | 100.000 65.620 134.380

Sub-total |

D+L pooled WMD | 100.000 65.620 134.380

---------------------+---------------------------------------------------

161-180

Lollo (2014) | -112.370 -162.671 -62.069

Lollo (2014) | -164.790 -216.355 -113.225

Sub-total |

D+L pooled WMD | -138.260 -189.627 -86.893

---------------------+---------------------------------------------------

Test(s) of heterogeneity:

Heterogeneity degrees of

statistic freedom P I-squared** Tau-squared

1-20 675.73 9 0.000 98.7% 2.4e+04

41-60 0.00 0 . .% 0.0000

161-180 2.03 1 0.154 50.8% 698.5175

** I-squared: the variation in WMD attributable to heterogeneity)

Significance test(s) of WMD=0

1-20 z= 0.83 p = 0.405

41-60 z= 5.70 p = 0.000

161-180 z= 5.28 p = 0.000

Figure 15 Subgroup Meta-Analysis Output on Creatine Kinase (u/l) by Duration Range of the Studies Period

Figure 16 Forest Plot of Subgroup Meta-Analysis on Creatine Kinase (u/l) by Duration of the Study Period
